# Research priorities for maintaining biodiversity’s contributions to people in Latin America

**DOI:** 10.14324/111.444/ucloe.000002

**Published:** 2019-08-08

**Authors:** Richard G. Pearson, Enrique Martínez-Meyer, Mercedes Andrade Velázquez, Mercedes Caron, Rogelio O. Corona-Núñez, Katrina Davis, América Paz Durán, Rodrigo García-Morales, Talya D. Hackett, Daniel J. Ingram, Rafael Loyola Díaz, Julián Lescano, Andrés Lira-Noriega, Yolanda López-Maldonado, Daniela Manuschevich, Alma Mendoza, Ben Milligan, Simon C. Mills, Darío Moreira-Arce, Luzma F. Nava, Vicencio Oostra, Nathan Owen, David Prieto-Torres, Clarita Rodríguez Soto, Thomas Smith, Andrew J. Suggitt, Camila Tejo Haristoy, Jorge Velásquez-Tibatá, Sandra Díaz, Pablo A. Marquet

**Affiliations:** 1 Centre for Biodiversity and Environment Research, Department of Genetics, Evolution and Environment, University College London, London, UK; 2 Departamento de Zoología, Instituto de Biología, Universidad Nacional Autónoma de México, Ciudad de México, Mexico; 3 Centro del Cambio Global y la Sustentabilidad AC, Villahermosa, Mexico; 4 Instituto Multidisciplinario de Biología Vegetal (IMBIV), Universidad Nacional de Córdoba (UNC), CONICET, Córdoba, Argentina; 5 Procesos y Sistemas de Información en Geomática, SA de CV. Calle 5 Viveros de Peten No. 18, Col. Viveros del Valle, Tlalnepantla, CP 54060, Edo. de Mex, Mexico; 6 Land, Environment, Economics and Policy Institute, University of Exeter Business School, Xfi Building, Rennes Drive, Exeter, UK; 7 Instituto de Ecología y Biodiversidad, Casilla 653, Santiago, Chile; 8 Department of Zoology, University of Oxford, Oxford, UK; 9 Instituto de Diversidad y Ecología Animal (IDEA), Universidad Nacional de Córdoba, Consejo Nacional de Investigaciones Científicas y Técnicas (CONICET), Córdoba, Argentina; 10 Instituto de Ecología, A.C. Carretera antigua a Coatepec 351, Col. El Haya, Xalapa, Veracruz, Mexico; 11 Department of Geography, Ludwig Maximilian University of Munich, Munich, Germany; 12 Universidad Academia de Humanismo Cristiano, Escuela de Geografía, Condell 343, Providencia. Santiago, Chile; 13 International Institute for Applied Systems Analysis (IIASA), Schloßpl. 1, Laxenburg, 2361, Vienna, Austria; 14 Institute for Sustainable Resources, University College London, London, UK; **Current address:** Global Water Institute, University of New South Wales, Sydney, Australia; 15 Department of Animal and Plant Sciences, University of Sheffield, Sheffield, UK; 16 Laboratorio de Estudios del Antropoceno, Facultad de Ciencias Forestales, Universidad de Concepción, Chile; 17 Organismal and Evolutionary Biology Research Programme, Research Centre for Ecological Change, University of Helsinki, Helsinki, Finland; 18 Departamento de Biología Evolutiva, Facultad de Ciencias, Museo de Zoología, Universidad Nacional Autónoma de México, México City, Mexico; 19 Centro de Estudios e Investigación en Desarrollo Sustentable, Universidad Autónoma del Estado de México, Toluca, Mexico; 20 Sustainability Research Institute, School of Earth and Environment, University of Leeds, Leeds, UK; 21 Department of Biology, University of York, York, UK; 22 Instituto de Conservación, Biodiversidad y Territorio, Facultad de Ciencias Forestales y Recursos Naturales, Universidad Austral de Chile, Valdivia, Chile; 23 Centro de Ciencia del Clima y la Resiliencia, Facultad de Ciencias Físicas y Matemáticas, Universidad de Chile, Santiago, Chile; 24 NASCA Conservation Program, The Nature Conservancy, Bogotá, Colombia; 25 Instituto Multidisciplinario de Biología Vegetal, CONICET and Universidad Nacional de Córdoba, Córdoba, Argentina; 26 Departamento de Ecología, Facultad de Ciencias Biológicas, Pontificia Universidad Católica de Chile, Chile; 27 Instituto de Ecología y Biodiversidad (IEB), Laboratorio Internacional en Cambio Global (LINCGlobal), Centro de Cambio Global UC (PUCGlobal), The Santa Fe Institute, and Centro de Ciencias de la Complejidad (C3), Universidad Autónoma de México, Mexico

**Keywords:** ecosystem services, environmental change, capacity building, investment in research, data availability, knowledge systems, governance

## Abstract

Maintaining biodiversity is crucial for ensuring human well-being. The authors participated in a workshop held in Palenque, Mexico, in August 2018, that brought together 30 mostly early-career scientists working in different disciplines (natural, social and economic sciences) with the aim of identifying research priorities for studying the contributions of biodiversity to people and how these contributions might be impacted by environmental change. Five main groups of questions emerged: (1) Enhancing the quantity, quality, and availability of biodiversity data; (2) Integrating different knowledge systems; (3) Improved methods for integrating diverse data; (4) Fundamental questions in ecology and evolution; and (5) Multi-level governance across boundaries. We discuss the need for increased capacity building and investment in research programmes to address these challenges.

## Introduction

Biodiversity contributes to people’s quality of life, for example, by pollinating crops, controlling pests, promoting soil fertility, and providing goods and aesthetic pleasure. Maintaining biodiversity to secure the supply of these benefits is crucial for ensuring human well-being, including through economic development and poverty alleviation [1]. We participated in a workshop held in Palenque, Mexico, 28–30 August 2018, that brought together 30 mostly early-career scientists working in different disciplines (natural, social and economic sciences) from across Latin America and the UK. Our aim was to identify research priorities for studying the manifold contributions of biodiversity to people and how these contributions might be impacted by environmental change. The workshop focussed on Latin America, which has particular challenges related to conserving globally significant biodiversity while addressing social and economic problems [2], but all of the points discussed will resonate with similar challenges in other regions of the world.

Here, we provide a summary of the key research priorities identified in the workshop.

Research priorities were identified through a series of break-out discussion groups followed by plenary discussions in which participants first identified a broad set of candidate questions, before iteratively paring the long list down and grouping them by topic. Discussions centred around key research questions that need to be answered to inform policy decision-making. We also discussed the feasibility of answering each question, and the funding and capacity building mechanisms that will be needed. Our list is by no means exhaustive and is subjective in so far as it is based on the expert opinion of those participating in the workshop, but we see particular value in this being the opinions of early-career researchers who will themselves push forward this research agenda over the coming decades. Our goal here is to share the overarching conclusions of our workshop with a view to stimulating future in-depth research into these important topics.

## Priority research questions

Five main groups of questions emerged, which we summarise below and in Table 1. A first topic centred around how the quantity and quality of data relating to biodiversity could be enhanced, and how those data could be made more widely available to diverse users. High quality baseline data relating to multiple dimensions of biodiversity – genetic, taxonomic, phylogenetic and functional – is often lacking and yet is fundamental to understanding responses to environmental change. We therefore identified a need to establish more rapid biodiversity assessment programmes, to strengthen long-term monitoring programmes, to use standardised collection protocols, and to use modern technologies such as eDNA and remote sensing to capture data. Moreover, although significant progress in data sharing has been achieved in recent years [e.g. through the Global Biodiversity Information Facility (GBIF)], data are too often inaccessible to relevant stakeholders. More activity in compiling large datasets (e.g. [3–6]) is needed, and as a community we need to incentivise data sharing, for instance, through promotions criteria that recognise contributions to shared repositories (e.g. [7]).

**Table 1.** Key areas for future research with example priority research questions..

**Enhancing the quantity, quality and availability of biodiversity data**
How can we accelerate the collection of biodiversity data?
How can we facilitate access to and sharing of ecological, environmental, and socially relevant data?
**Integrating different knowledge systems**
Does incorporating different world views result in better management of biodiversity and the associated benefits for humans?
How do power imbalances influence the integration of different values in the governance of ecosystem services?
**Improved methods for integrating diverse data**
How can we best integrate data from various sources and across different spatial and temporal scales?
How can we improve the uptake of methods that consider uncertainty, ecological interactions, non-linearity and synergistic effects?
**Fundamental questions in ecology and evolution**
How does the distribution of genetic variation across the genome and across species’ geographical ranges determine capacity for evolutionary adaptation to rapid anthropogenic change?
How sensitive are ecological communities to perturbation, how robust are they to species loss, and what aspects of the community determine this?
**Multi-level governance across boundaries**
How can we conserve, restore or enhance ecosystems and biodiversity, and associated benefit and detriment flows, that extend across local or national boundaries?
How can (or should) nested scales of governance (local, national, international; public, private) be coordinated and reformed to enhance benefits to people from biodiversity and ecosystems?

A second set of questions focussed on the challenge of integrating different world views and value systems. The Intergovernmental Science-Policy Platform on Biodiversity and Ecosystem Services (IPBES) has adopted a framing that uses the notion of ‘nature’s contributions to people’ (NCP; [8]), which fully includes, but goes beyond, that of ecosystem services. The NCP approach recognises the role that culture plays in defining links between people and nature, and incorporates local and traditional knowledge [9] alongside that of Western science. This raises important questions about how exactly different world views can be integrated in biodiversity studies and whether doing so results in better management of benefits and detriments to people. Central to these questions will be issues relating to power imbalances, as power dynamics strongly influence what aspects of biodiversity are prioritised for research and are particularly relevant to the quality of life of marginalised people.

Our third category of questions included diverse issues relating to the need for improved methods of analysis. As increasing quantities of data are made available from different sources, at varying spatial and temporal scales, and relating to diverse phenomena in natural and social sciences, there is a need for more transdisciplinary methods that can help us to make sense of these rich sources of information. Such methods will need to incorporate robust ways to deal with uncertainty, and must allow for the consideration of complex, non-linear and delayed responses resulting from ecological interactions (e.g. [10]) and synergies between threats (e.g. [11]).

A fourth set of questions focussed on areas of research that are currently hot topics in ecology and evolutionary biology, and that are deemed of key importance for ensuring adequate management of biodiversity and the sustainability of its contributions to people. A wealth of questions was discussed relating to the responses of individuals, populations, species and communities to environmental perturbations, and the functional responses that will define the benefits that people derive from nature. In some cases the questions related to classic debates (such as concerning the relationship between diversity and stability; [12]) and there was scepticism that they would be answered in the next 5 to 10 years. However, several questions were viewed as both pressing in an applied sense and also feasible to answer in light of new methods, particularly with regard to generating a more mechanistic understanding of how biodiversity responds to anthropogenic change.

A final set of questions concerned governance challenges, especially relating to the transboundary management of biodiversity and ecosystems, and the links between public and private sectors. Transboundary management is essential given the globalised or transnational nature of environmental change drivers, and the spatial misalignment of governance boundaries and ecosystems. This also relates to the need for biodiversity datasets that extend across multiple countries and are widely available in standardised formats, in line with the first category of questions that we identify above. Governance reforms will be necessary to meet each country’s international commitments, such as under the Convention on Biological Diversity and through the Sustainable Development Goals, yet further research is needed as to how collective decision making, institutions and norms can or should mediate, allocate or otherwise influence flows of benefits to people from ecosystems and biodiversity.

## What is needed to answer the questions?

Latin America will play an important part in the future of global change at the planetary scale; for example, deforestation in the Amazon and the melting of Patagonia’s glaciers will strongly affect the hydrological cycle and climate across the Americas and possibly beyond. Yet most nations in Latin America have biodiversity and ecosystem research low down their agendas. Enhancing human well-being requires that we increase efforts to protect and restore the many ways in which biodiversity contributes to people and ensure that those contributions are long lasting and accessible to all. In order to foster and accelerate research that will address the key questions that we have identified, we recommend: (1) A focus on capacity building to educate transdisciplinary researchers, increase transboundary training, meet training needs in less well-served regions, and retain young researchers in the region; and (2) Investment in research programs that are transdisciplinary, support international collaboration across the region and beyond (such as through the Newton Fund that funded our workshop), are long term, and are of sufficient magnitude to realistically address these challenging research needs.

## Data Availability

No further data were used in addition to referenced works.
